# The Impact of Employee Service Competence on Gen Z Food Consumption Decisions: The Moderating Role of OMO Contexts

**DOI:** 10.3390/foods14162793

**Published:** 2025-08-11

**Authors:** Wenyan Yao, Mohd Anuar Arshad, Mengjiao Zhao, Chenshu Yu

**Affiliations:** 1 College of Business, Nanning University, Nanning 530200, China; yaowenyan@unn.edu.cn; 2 School of Management, Universiti Sains Malaysia, Gelugor 11800, Malaysia; rubymengjiao@gmail.com

**Keywords:** employee service competence, Generation Z, repurchase intention, customer satisfaction, brand trust, Online-Merge-Offline, foodservice enterprises

## Abstract

As Generation Z gradually becomes the dominant consumer group, the catering industry, as a critical sector affecting people’s livelihood, warrants an in-depth investigation into its consumption decision mechanisms. This study, grounded in the online–merge–offline (OMO) context, empirically examines the impact mechanism of frontline employee service competence on the repurchase decisions of Generation Z consumers in the foodservice sector, while testing the mediating roles of customer satisfaction and brand trust, as well as the moderating effect of the OMO scenario. Data were collected via a survey of 326 Generation Z customers who consumed in integrated OMO dining environments. Partial least squares structural equation modeling (PLS-SEM) was employed for the data analysis. The findings reveal that frontline employee service competence significantly and positively influences consumer repurchase intention and customer satisfaction, but does not have a significant effect on brand trust. Customer satisfaction fully mediates the relationship between employee service competence and repurchase decisions, whereas brand trust, despite having the strongest direct effect on repurchase intention, is predominantly shaped by systemic factors such as food safety and supply chain transparency, rendering its mediating pathway non-significant. Furthermore, the OMO context does not exhibit a significant moderating effect between employee service competence and customer satisfaction, nor between employee service competence and brand trust, reflecting that the current digital integration has yet to effectively address Generation Z’s core needs for privacy protection and emotional resonance. This study elucidates the “contact–cognition–behavior” pathway by which service competence influences consumer decision-making through satisfaction, while clarifying the systemic formation mechanism of brand trust. Based on these results, it is recommended that enterprises prioritize emotional service training for frontline employees to enhance satisfaction, build brand trust through ingredient traceability systems, and optimize OMO scenario design to better align with Generation Z’s expectations for emotional interaction.

## 1. Introduction

Against the dual backdrop of digital transformation and the shifting landscape of consumer sovereignty, Generation Z (born between 1995 and 2009), as true “digital natives,” has emerged as a core consumer segment. According to the *2020 Gen Z Consumer Attitudes Insight Report*, Generation Z has a global population of approximately 1.85 billion, accounting for 24% of the world’s total population. Meanwhile, McKinsey’s *2024 Global Consumer Insight Report* [1] reveals that Gen Z now contributes 40% of global consumption growth. In the Chinese market, their annual consumption expenditure has surpassed RMB 4.2 trillion, with their share in the foodservice sector exceeding 39%. Furthermore, the *2023 Investment Strategy Report on China’s Food and Beverage Industry* indicates that 62% of Generation Z consumers develop brand trust (BT) through short videos and livestreaming platforms, while 73% abandon purchases due to delayed service responses [2]. This cohort’s reliance on digital services coupled with their demand for emotionally engaging experiences is driving a fundamental transformation in service delivery models within the foodservice industry [3]. Foodservice enterprises are progressively transitioning from a traditional, segmented online-offline operational model toward an integrated online–merge–offline (OMO) approach [4].

Within this context, frontline employee service competence (ESC), as a critical touchpoint in the service delivery process, has been empirically shown to significantly enhance consumer satisfaction (CS) [3]. For instance, although 85% of service firms have deployed AR and smart terminal technologies, only 32% of frontline employees can proficiently operate these devices, and 41% of consumers have encountered issues such as inability to redeem online coupons offline and loss of service records across channels, according to data from the China Chain Store and Franchise Association [5]. Prior studies have established that CS directly influences repurchase intentions (RI) in the catering sector [6]. However, RI holds significant importance for the development of foodservice enterprises. A report from a U.S.-based strategic consultancy reveals that a 5% increase in customer retention can boost profits by 25% to 85% [7], as acquiring new customers costs five to six times more than retaining existing ones, with loyal customers contributing over 65% of firm revenues [8].

Although abundant research has confirmed the positive relationship between ESC and RI, the moderating role of the OMO model—whose penetration rate in the catering industry exceeds 53.9%—remains empirically underexplored [9]. Moreover, Generation Z’s unique blend of technological savviness and emotional needs challenges traditional theoretical frameworks in explaining their decision-making processes [10]. To address these gaps, this study adopts the service-dominant logic as a theoretical foundation to investigate:

**RQ1:** How does ESC influence Generation Z’s RI through the dual mediating effects of CS and BT?

**RQ2:** Does the OMO context strengthen the effect of ESC on CS and BT?

Accordingly, this study positions ESC as the independent variable, examining its impact on Generation Z consumers’ RI in foodservice enterprises. CS and BT are incorporated as mediating variables, while the OMO context serves as a moderating variable. This framework aims to provide an in-depth understanding of how ESC shapes RI among Generation Z consumers.

## 2. Literature Review

### 2.1. Employee Service Competence

From an industrial perspective, Spencer (1993) defined competence as the underlying personal characteristics of individuals that result in superior performance within a given context [11]. Rainsbury et al. (2002) further conceptualized competence as comprising two components: hard skills, which pertain to technical and administrative domains and are generally associated with the acquisition of knowledge; and soft skills, which relate to personal and interpersonal behaviors [12]. In the context of frontline service employees, Yu-Chi Wu et al. (2015) [13] proposed that service competence consists of two primary dimensions essential for effective task execution during customer interactions: product expertise and interpersonal competence. Product expertise refers to the knowledge, technical skills, and domain-specific competencies required to address customer needs, while interpersonal competence involves the ability to communicate and interact effectively with customers.

Moreover, prior studies have highlighted the critical role of empathic understanding in frontline service work. Empathic competence refers to the employee’s ability to accurately perceive and respond to customers’ emotional states, thereby influencing customer evaluations and behavioral intentions [14]. Employees with high empathic ability are better equipped to infer customers’ emotional experiences and respond with genuine concern, thereby enhancing the perceived emotional quality of service [15]. As the integration of online and offline channels (OMO) becomes increasingly prevalent, human–technology interactions have been incorporated into frontline service contexts. To enhance the customer experience and reduce friction in service encounters, the definition of frontline employee competence has expanded beyond traditional service capabilities to include digital service competence, such as the ability to facilitate online–offline integration and manage remote interactions effectively [16]. As noted by Laasch et al. (2023) [17], although specific individual competencies are highly valuable, they are insufficient on their own for frontline employees to deliver satisfactory service to customers. This underscores the need for an integrated approach to frontline service competence.

Drawing upon the extant literature, this study conceptualizes employee service competence in OMO contexts as a multidimensional construct encompassing product expertise, communication and interaction competence, empathic understanding, and digital service competence.

### 2.2. The Direct Relationship Between Employee Service Competence, Consumer Satisfaction, Brand Trust, and Repurchase Intention

Repurchase intention refers to a customer’s inclination to engage in subsequent purchases following a prior consumption experience, while also sharing their positive experiences with others and forming lasting memories [18]. Prior research consistently indicates that higher levels of CS significantly increase the likelihood of repurchase behavior [6]. Employee service competence, defined as the frontline employees’ ability to deliver services that meet or exceed customer expectations [19], has been identified as a critical factor influencing customer decision-making processes [20]. Specifically, the product expertise of frontline staff directly shapes CS, which in turn influences their purchasing decisions [21]. Employees’ communication and interaction competence enable them to respond quickly to customer needs and provide personalized services, helping to build emotional connections, enhance customer trust, and ultimately encourage repeat purchases. [22]. Additionally, affective interaction competence enhances customers’ perceived value of the service encounter, which positively contributes to their RI [23]. Based on the foregoing, this study proposes the following hypothesis:

**H1.**  *There is a positive relationship between ESC and RI*.

Customer satisfaction is defined as a psychological state of contentment experienced by consumers when the product or service received aligns with or exceeds their expectations [24]. ESC, referring to the capacity of frontline staff to deliver services that fulfill customer needs [19], plays a pivotal role in determining the level of CS [25,26]. Employees with high service capabilities directly impact service reliability (e.g., accurate execution of processes) and service speed (e.g., prompt response to needs). These factors help reduce perceived risk and build customer trust, thereby enhancing the overall customer experience [27]. When the service provided meets consumer expectations, customer satisfaction is achieved [28]. Moreover, empirical research indicates that frontline employees with superior service competence can foster customer trust and mitigate perceived risk, which further enhances CS [29]. Accordingly, this study proposes the following hypothesis:

**H2.**  *There is a positive relationship between ESC and CS*.

Brand trust refers to consumers’ confidence in a brand’s reliability and their willingness to believe that the brand will fulfill its promises. When consumers face risk, this trust manifests as an expectation that the brand will deliver positive outcomes [30]. Employees are a key source through which consumers perceive and develop trust in a brand [31]. Andrews et al. (2022), in their investigation of frontline employee’s ability and willingness to deliver brand-aligned service, found that employees significantly influence customers’ trust in the brand [32]. Similarly, Huang et al. (2023) demonstrated that ESC has a direct effect on customers’ BT through their influence on the overall service experience [31]. This effect is particularly pronounced in high-contact service sectors, where employees’ behaviors and competencies contribute significantly to shaping brand image and consequently customer trust [33]. As ESC enhances perceived service quality, it positively affects the degree of trust consumers place in the brand [34]. Accordingly, the following hypothesis is proposed:

**H3.**  *There is a positive relationship between ESC and BT*.

CS has been widely recognized as a key determinant of revisit behavior in the foodservice enterprises [35]. When diners have a favorable dining experience, they are more likely to recommend the restaurant to others, engage in positive word-of-mouth communication, and demonstrate loyalty to the brand [21]. Satisfaction is, thus, regarded as a critical success factor for foodservice businesses [36]. Satisfied customers are more likely to return, whereas dissatisfied customers tend to hesitate before making repeat purchases [37]. Therefore, maintaining high levels of CS is essential for ensuring customer retention and encouraging repurchase behavior, especially in high-contact service contexts such as restaurants [28]. Prior studies further support this relationship. For example, Wirakananda (2021) found that CS plays a pivotal role in shaping RI [38]. Similarly, Han and Jung (2021) confirmed a significant positive influence of satisfaction on consumers’ willingness to repurchase [39], a finding echoed by Damanik and Yusuf (2021), who emphasized the strong association between CS and RI [40]. Accordingly, the following hypothesis is proposed:

**H4.**  *There is a positive relationship between CS and RI*.

Brand trust has been demonstrated to play a key role in consumers’ purchasing decisions. It not only enhances consumers’ perceptions of product safety and reliability but also strengthens their confidence in decision-making, thereby increasing the likelihood of purchase behavior [41]. According to Cardoso et al. (2022), BT contributes to the cultivation of consumer loyalty, reinforces RI, and deepens the emotional connection between consumers and the brand [41]. Empirical studies conducted in specific consumption contexts further support this assertion. For instance, Oktavia et al. (2024) revealed that within the Shopee mobile application, BT exerts a significant and positive impact on consumer purchase decisions—the higher the level of trust, the more likely consumers are to make purchases via the platform [42]. Similarly, research on various brand contexts consistently demonstrates that BT is a critical determinant in consumer brand selection. In addition, Tyagita et al. (2024) highlighted that BT exerts both direct and indirect effects on customer loyalty [43], ultimately influencing consumers’ behavioral intentions. When consumers trust a brand, they are more likely to maintain loyalty, as the post-purchase satisfaction reinforces their intention to repurchase and strengthens long-term commitment. Drawing on these findings, the following hypothesis is proposed:

**H5.**  *There is a positive relationship between BT and RI*.

### 2.3. The Mediating Role of Comsumer Satisfaction and Brand Trust and the Moderating Role of Perceived OMO Scenario

CS is defined as the result of a comparative evaluation between consumers’ perceptions of the performance of a product or service and their pre-consumption expectations [44]. Extensive research has established that ESC significantly enhances CS, particularly in the restaurant industry, where service encounters are frequent and highly personalized [45,46]. Furthermore, CS has been shown to influence various downstream behavioral outcomes, including customer loyalty and RI [40]. High levels of satisfaction may lead not only to increased repurchase behavior but also to consumers’ willingness to pay a price premium. Moreover, CS can reduce decision complexity by fostering emotional commitment, thereby minimizing consumers’ need for extensive information searches during future purchase decisions [47]. Research has shown that in service-intensive industries (such as hospitality), customer satisfaction fully mediates the relationship between ESC and RI [48]. This implies that the effect of ESC on RI is channeled primarily through the satisfaction customers derive from the service encounter. Based on this prior literature, the following hypothesis is proposed:

**H6.**  *CS mediates the relationship between ESC and RI*.

BT refers to a consumer’s confidence in a brand’s reliability and its intention to fulfill promises, particularly under conditions of uncertainty [49]. In high-contact service contexts, the service capabilities of frontline employees directly influence consumers’ assessment of a brand’s ability to deliver on its commitments [23]. BT serves as a full mediator between prior consumption experiences and brand loyalty, with service quality emerging as a key antecedent [50]. Moreover, employees’ interpersonal and communicative competencies have been shown to strengthen consumers’ trust in the brand’s customer-centric orientation [51]. Prior studies have consistently found that consumers’ trust in a brand significantly and positively affects their purchase decisions—higher levels of brand trust are associated with an increased likelihood of purchasing [42]. Supporting this, Ichou and Manar (2024) empirically confirmed a significant positive association between BT and consumer purchase behavior, emphasizing the pivotal role of trust in enhancing RI and elevating the probability of repeat purchasing [52]. Based on this, this paper proposes the following hypothesis:

**H7.**  *BT mediates the relationship between ESC and RI*.

The foodservice enterprises context refers to a deliberately designed consumption environment intended to shape customer perceptions and dining satisfaction during service encounters [53]. In recent years, online–merge–offline (OMO) service settings have emerged as an advanced service model that integrates data-driven operations, process optimization, and multichannel coordination to enhance customers’ digital experiences [54]. This hybrid model enables consumers to complete tasks such as reservations, ordering, and payments online while receiving personalized and immersive services offline [55].

For frontline employees, such dual-channel integration necessitates proficiency in both digital interface management and real-time in-person service delivery. Members of Generation Z, having matured in a digitally saturated environment, demonstrate substantially higher acceptance of digitalized consumption models compared to previous generational cohorts [56]. Consequently, OMO-enabled experiences have become increasingly influential in shaping Gen Z’s service expectations and brand perceptions.

Prior research has shown that the implementation of OMO service environments can augment employees’ effectiveness and improve CS [57]. By automating routine operational tasks through online systems, OMO settings allow frontline staff to reallocate their cognitive and emotional resources toward high-value interactions such as personalized communication and conflict resolution [58]. Furthermore, data-enabled personalized services are found to significantly increase affective satisfaction and subsequently enhance BT [59,60].

Based on the foregoing discussion, this study proposes the following hypotheses:

**H8.**  *OMO moderates the relationship between ESC and CS, which becomes higher when OMO is high*.

**H9.**  *OMO moderates the relationship between ESC and BT, which becomes higher when OMO is high*.

**Figure 1:**
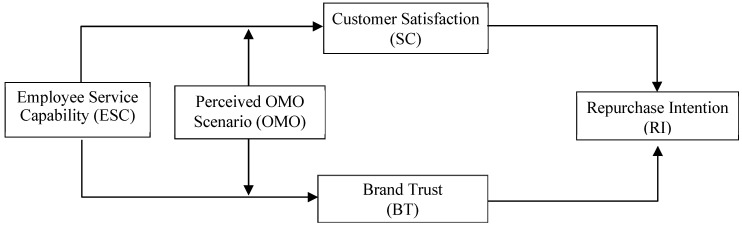
Research framework.

A research framework illustrating the proposed relationships is presented in Figure 1.

## 3. Methodology

### 3.1. Questionnaire Design

The questionnaire was divided into two sections. The initial segment of the study was designed to procure exhaustive demographic data concerning the respondents, including respondents’ gender, age, educational attainment, and daily consumption habits. These demographic variables were used to describe the characteristics of the sample population. The second section assessed several latent constructions, including ESC, CS, BT, perceived OMO scenarios, and RI. The present study adopts a conceptualization that has been validated for application in the Chinese context and which is supported by extant literature. The concepts under discussion are derived from the fields of organizational behavior and consumer behavior, and are adapted to fit the specific characteristics of the food consumption industry in an OMO scenario.

Employee service capability (ESC): This construction was operationalized using four subdimensions: product expertise, communication and interaction competence, empathic understanding, and digital service competence. Product expertise was measured using a three-item scale developed by Wu et al. [13], assessing the degree to which respondents perceived employees to possess comprehensive knowledge about the food and beverages provided. Communication and interaction competence was measured with a three-item scale, also adopted from Wu et al. [13], capturing the employees’ ability to effectively communicate and understand customer needs. Empathic understanding was assessed using a three-item scale from Delcourt et al. [61], evaluating employees’ ability to establish emotional connections and enhance consumer satisfaction through empathetic engagement. Digital service competence was measured with a three-item scale from Voorhees et al. [62], focusing on the perceived ability of employees to utilize digital tools to improve service efficiency and experience continuity.

Customer satisfaction (CS): This was assessed using a five-item scale developed by Stevens et al. [63], capturing the overall satisfaction of respondents with the dining experience and quality of food consumed.

Brand trust (BT): This was measured using a three-item scale proposed by Chaudhuri and Holbrook [64], evaluating the extent to which respondents trusted the restaurant brand to deliver reliable and consistent value.

Perceived OMO scenarios: This was measured using a seven-item scale developed by Zhang et al. [29], capturing respondents’ perception of the integration between online and offline services in OMO settings. Notably, the item “The environment is comfortable and pleasant” not only reflects the physical comfort of the dining space but also conveys respondents’ overall perception of the coordination between digital interfaces and human assistance, representing the holistic service experience in OMO contexts.

Repurchase intention (RI): This was measured using a four-item scale adapted from Yuliantoro et al. [65], assessing respondents’ willingness to repurchase and recommend the restaurant to others.

### 3.2. Participants and Procedure

This study employed purposive sampling to select respondents from consumers dining at restaurants. The inclusion criteria required participants to be born between 1995 and 2010 and to have experienced dining at restaurants featuring an integrated online-to-offline (OMO) service environment at least once. Exclusion criteria comprised individuals born in 1994 or earlier, those born in 2011 or later, and consumers who had no experience with OMO dining scenarios.

To ensure the quality of questionnaire responses, the researchers first contacted the restaurant managers and initiated the survey only after obtaining permission. Upon entering the restaurant, the researchers asked potential respondents about their willingness to participate. Once consent was confirmed, the researchers briefly explained the purpose of the study and obtained signed informed consent forms. For respondents who were minors during the period of 2007–2010, informed consent was obtained from their parents. All questionnaires were collected anonymously to protect the privacy of the respondents.

Following Hair’s recommendation that the minimum sample size should be at least five times the number of observed variables [66], and given that the survey instrument consisted of 31 items, the minimum sample size calculated was 155. Considering an estimated response rate of approximately 70% based on similar studies, the target sample size was set at 222. Data collection was conducted between February and April 2025 at two of China’s most representative dining establishments, Haidilao and Mixue. A total of 360 customers participated in the survey, yielding 326 valid responses, corresponding to a response rate of 90.6%. Demographics of the respondents are summarized in Table 1.

### 3.3. Partial Least Squares Structural Equation Modeling

In this study, partial least squares structural equation modelling (PLS-SEM) was utilized as the statistical analysis method. PLS-SEM has been shown to be more effective than CB-SEM in dealing with multiple relationships in the model. This is due to its ability to combine a principal component analysis with ordinary least squares regression, making it particularly suitable for research situations with non-normally distributed data or small sample sizes [67]. The present study was analyzed using SmartPLS 4.1 software, an excellent programme for the analysis of non-normal data and small samples, with the potential to effectively support the objectives of this study [68].

PLS-SEM is comprised of two core components. Firstly, the measurement model is utilized for the assessment of the relationship between observed variables and their underlying constructs. Secondly, the structural model is employed for the revelation of the path relationships between the constructs. The SmartPLS software was utilized to estimate the path coefficients, thereby facilitating the verification of the reliability and validity of the models.

#### 3.3.1. Evaluation Criteria for Measurement Models

Common method bias (CMB) is a systematic error that may arise from the fact that all data come from the same respondent when data are collected using a single method or source [69]. In order to test the CMB problem, the present study employed a one-way Harman test to assess the risk of bias through an exploratory factor analysis. When the results of the analysis demonstrated a single factor, or a solitary factor that accounted for over 50% of the explained variance (based on an unrotated matrix), this indicated a significant common method bias problem [70].

The assessment of measurement models necessitates the implementation of reliability and validity tests. In the context of reliability testing, four primary metrics are evaluated: outer loading (OL), Cronbach’s alpha (CA), composite reliability (CR), and average variance extracted (AVE). OL reflects the strength of the correlation between observed indicators (items) and their corresponding latent constructs [71]. CA evaluates internal consistency reliability based on the inter-correlations among items [72]. CR, calculated based on standardized loadings, is another measure of construct reliability [73]. AVE refers to the average amount of variance in the indicators that is explained by the latent construct, and it is used to assess convergent validity [74,75]. In accordance with the stipulated criteria of the evaluation framework, the values of OL [71], CA [72], and CR [73] are required to exceed 0.70, while the value of AVE is expected to surpass 0.5 [74,75].

Discriminant validity is defined as the extent to which a construct is theoretically clearly distinguishable from other constructs [76]. This concept is employed to evaluate the accuracy of a measurement tool in quantifying the intended construct, as opposed to quantifying something else. The present study finds the topic to be of relevance [77]. Discriminant validity is, therefore, a crucial part of measurement modelling. Commonly used assessment methods include the heterogeneity-to-monogeneity ratio (HTMT), the Fornell–Larcker criterion, and cross-loading analyses [78]. In this study, the discriminant validity of the model was confirmed by using the Fornell–Larcker criterion.

#### 3.3.2. Evaluation Criteria for Structural Models

The primary focus of a structural model assessment is the evaluation of path coefficients and their associated statistical significance tests. Furthermore, the R^2^ value is utilized to ascertain the extent to which the predictor variables explain the variance of the endogenous variables, thereby reflecting the strength of the explanatory power of the model [79]. The value of R^2^ ranges from 0 to 1, with higher values indicating stronger explanatory power [80]. According to Cohen’s rule of thumb [81], R^2^ values of 0.26, 0.13, and 0.02 are indicative of strong, moderate, and weak levels of explanation, respectively.

Here, f^2^ is used to measure the effect size, i.e., the extent to which each independent variable affects the outcome variable, and provides a strong basis for understanding the strength of the relationship between the variables [82]. According to Cohen’s suggestion [81], f^2^ values of 0.02, 0.15, and 0.35 indicate small, medium, and large effects, respectively. $Q^{2}$ is then used to assess the predictive power of the model [83]. When $Q^{2}$ is greater than 0, it indicates that the model has predictive relevance; if it is less than 0, it indicates insufficient predictive ability [84]. These assessment criteria help to comprehensively test the validity of the model, thereby enhancing the reliability of the research results [85].

## 4. Results and Analysis

### 4.1. Common Method Variance

In this study, common method bias was assessed using a one-way Harman test, which showed that a single factor explained only 41.175% of the total variation, which is lower than the generally accepted threshold of 50%, suggesting that common method bias had a small impact on the results of this study.

### 4.2. Measurement Model Assessment

#### 4.2.1. Convergent Validity

**Figure 2:**
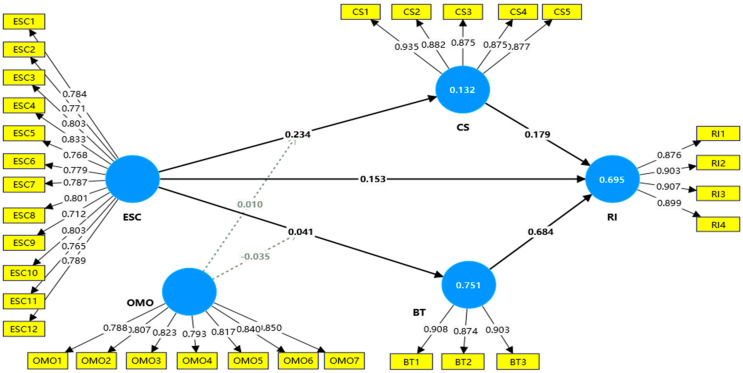
Measurement Model.

Based on the criteria in Section 3.3.1, this study assessed the internal consistency and convergent validity of the constructs by examining their factor loadings with reliability indicators. Table 2 summarizes the loadings of the constructs’ indicators, and the results show that all indicators are above 0.7, indicating good internal consistency and convergent validity. The modified measurement model is shown in Figure 2.

As illustrated in Table 3, the results of the reliability tests of the structural models are presented, including Cronbach’s alpha, CR, and AVE values. In accordance with the criteria delineated in Section 3.3.1, Cronbach’s alpha values for the five constructs range from 0.876 to 0.943, thereby exceeding the benchmark of 0.70 and indicating strong internal consistency.

For CR, the values fall between 0.924 and 0.950, which are all above the acceptable level of 0.70, demonstrating satisfactory overall reliability. Regarding AVE, the values range from 0.614 to 0.803, all above the minimum threshold of 0.50, indicating robust convergent validity and internal consistency.

#### 4.2.2. Discriminant Validity

In this study, discriminant validity was assessed using the Fornell–Larcker criterion (Fornell and Larcker, 1981) by comparing the square root of the AVE of each construct with its correlation coefficient with other constructs [86]. A construct is considered to have good discriminant validity if its AVE square root is greater than its maximum correlation with all other constructs [87]. Specifically, when the AVE square root is higher than the non-diagonal elements of the corresponding rows and columns in the correlation matrix, this signifies that the correlation between the construct and its measures is higher than its correlation with other constructs [67].

As demonstrated in Table 4, the AVE square roots of BT (0.895), RI (0.896), CS (0.889), ESC (0.783), and OMO (0.817) exceed the other correlation coefficients in their respective ranks within the correlation matrix. This finding suggests that there is adequate discriminant validity among the constructs.

### 4.3. Final Path Coefficients

**Figure 3:**
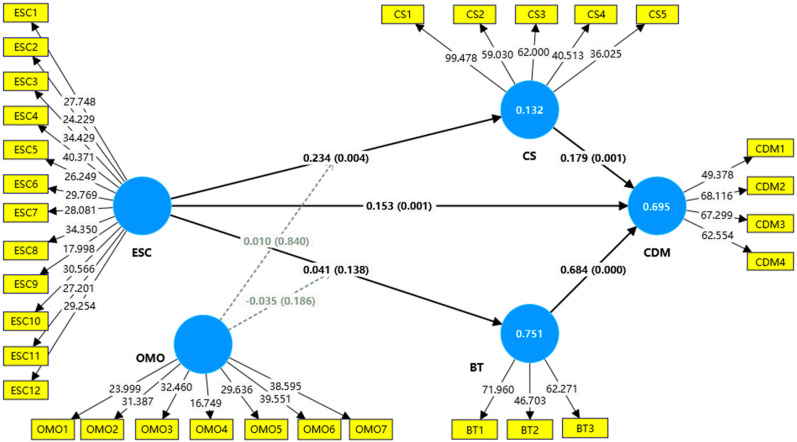
Structure model test.

Within the structural model, ESC was designated as the independent variable, while RI was identified as the dependent variable. CS and BT were recognized as the mediating variables, and OMO was positioned as the moderating variable. The bootstrapping analyses were conducted using SmartPLS, with significance tests set at the 0.05 level. In the present study, a total of nine hypotheses were put to the test, incorporating five direct effects, two mediating effects, and two moderating effects. The results of the tests are displayed in Figure 3 and Table 5.

As shown in Table 5, there is a significant positive relationship between ESC and RI (β = 0.153, T = 3.319, *p* < 0.05), thereby supporting hypothesis 1 (H1). This finding indicates that ESC has a positive impact on consumers’ intention to repurchase. Moreover, ESC is positively associated with CS (β = 0.234, T = 2.850, *p* < 0.05), supporting hypothesis 2 (H2). This suggests that ESC plays an important role in enhancing CS within the foodservice sector. However, no significant relationship can be observed between ESC and BT (β = 0.041, T = 1.483, *p* > 0.05), meaning hypothesis 3 (H3) is not supported. This implies that ESC does not significantly contribute to enhancing trust in restaurant brands.

Furthermore, CS can be seen to be positively associated with RI (β = 0.179, T = 3.437, *p* < 0.05), supporting hypothesis 4 (H4). Similarly, BT also exhibits a significant positive effect on RI (β = 0.684, T = 15.431, *p* < 0.05), supporting hypothesis 5 (H5). These findings suggest that enhancing CS and BT can substantially strengthen consumers’ RI.

Hypothesis 6 (H6), which posits the mediating effect of CS between ESC and RI, is also supported (β = 0.076, T = 2.709, *p* < 0.05). This indicates that improved ESC enhances CS, which in turn positively influences RI. However, hypothesis 7 (H7) is not supported, as the mediating effect of BT between ESC and RI is not significant (β = 0.028, T = 1.450, *p* > 0.05), suggesting that BT does not serve as a mediator in the relationship between ESC and RI.

Lastly, the moderating effects of the OMO consumption scenario were tested. The interaction effect of OMO on the relationship between ESC and CS is not significant (β = 0.002, T = 0.192, *p* > 0.05), providing no support for hypothesis 8 (H8). Likewise, the moderating effect of OMO on the relationship between ESC and BT is also not significant (β = −0.024, T = 1.333, *p* > 0.05), rejecting hypothesis 9 (H9). These results imply that OMO consumption scenarios do not moderate the effects of employee service capability on customer satisfaction or brand trust.

### 4.4. Effect Size and Predictive Power of the Model

As illustrated in Table 6, the extent to which exogenous constructs influence the accuracy and relevance of model predictions is demonstrated. The present analysis appraises the relative contribution of the constructs to the RI predictions and the magnitude of the role of ESC in predicting CS and BT.

The results indicate that the effect sizes of ESC and CS on RI are f^2^ = 0.062 and f^2^ = 0.091, respectively—both falling within the range of 0.02 to 0.15—suggesting small effects. In contrast, the effect size of BT on RI is f^2^ = 1.251, far exceeding the threshold of 0.35, indicating a very large effect. The effect of ESC on CS is also small (f^2^ = 0.051), while its effect on BT is negligible (f^2^ = 0.005), well below the threshold of 0.03. Overall, the f^2^ values range of 0.005 to 1.251 indicates that the pathways identified in the study are statistically significant and also meaningful in the context of the study.

The predictive relevance of the model as a whole was determined to be Q^2^ = 0.545, with the predictive relevance values of ESC for CS and BT being Q^2^ = 0.099 and Q^2^ = 0.589, respectively. This finding indicates that the model exhibits satisfactory predictive ability for all endogenous constructs.

Furthermore, the R^2^ values were utilized to evaluate the extent to which the predictor variables explained the variance of the endogenous variables. The findings indicated that the R^2^ of RI was 0.692, CS was 0.124, and BT was 0.748, suggesting that the model exhibited a substantial explanatory capacity for the endogenous variables and exerted a notable explanatory effect.

The goodness-of-Fit (GoF) was used to assess the overall performance of both the measurement model and the structural model. The GoF value in this study was calculated to be 0.619. According to the criterion proposed by Wetzels et al. (2009) [88], a GoF value ≥ 0.36 indicates a very strong model fit. In addition, the standardized root mean square residual (SRMR) was used to assess the model fit. The SRMR value for the current model is 0.051. According to Hu and Bentler (1998), SRMR values below 0.10 are considered acceptable [89]. Furthermore, the normed fit index (NFI) is 0.907, exceeding the threshold of 0.90 as recommended by Bentler and Bonett (1980) [90], indicating a good model fit.

The results of the aforementioned tests are displayed in Table 6.

## 5. Discussion

Focusing on service-dominant logic (SDL), this study explores how employee service competence affects Gen Z’s repurchase intention through consumer satisfaction and brand trust, and examines whether the OMO context has a moderating role in this process. The OMO model has been widely adopted in the restaurant industry, and Generation Z consumers also exhibit the co-existence of technological sensitivity and emotional appeal, so it is relevant to explore the relationship between these variables.

The study tested nine hypotheses, five were valid and four were not. In the upcoming discussion, this study refers to the previous literature and examines the findings through different perspectives to understand the relationship between frontline employee’s service competence, consumer satisfaction, brand trust, perceived OMO scenarios, and repurchase intention in catering companies by exploring the relationship between these variables, so as to provide practical suggestions for the operation and management of catering companies.

### 5.1. The Direct Relationship Between ESC, CS, BT, and RI

Hypothesis 1 (H1) proposed a positive relationship between ESC and RI. This was confirmed (β = 0.153, T = 3.319, *p* < 0.05), indicating that ESC positively influences Generation Z consumers’ RI. Prior research corroborates this finding [3], as employees’ product expertise enhances CS, which in turn fosters RI [21]. Additionally, employees’ communication and interpersonal skills, including empathetic engagement, enable prompt response to customer needs and personalized service delivery, reinforcing trust and repeat purchase behaviors. Empathetic interactions also bolster perceived service value, thereby enhancing RI [22].

Hypothesis 2 (H2) posited a positive effect of ESC on CS, which was supported (β = 0.234, T = 2.850, *p* < 0.05). Employees with high service competence efficiently execute customer instructions, offer useful recommendations, and perform tasks reliably, thereby enhancing the service experience and shaping positive first impressions [27]. When service meets or exceeds consumer expectations, CS is achieved [28]. These results suggest that enhancing ESC can improve both operational efficiency and CS in the foodservice sector—offering managerial implications for targeted training interventions.

Hypothesis 3 (H3), which asserted that ESC would positively affect BT, was not supported (β = 0.041, T = 1.483, *p* > 0.05). This aligns with previous findings that BT is largely rooted in structural assurances such as food safety transparency and supply chain traceability—not single transaction experiences [91]. In the foodservice context, trust depends on factors such as consistent product quality, credible user reviews, and third-party certifications, especially in crisis management, rather than frontline service alone [92].

H4 examined the relationship between CS and RI. The results demonstrated a significant positive effect of CS on RI (β = 0.179, T = 3.437, *p* < 0.05). This finding is consistent with existing research that suggests a positive customer experience at a restaurant is associated with an increased likelihood of positive recommendations, the dissemination of positive evaluations, and higher loyalty [21]. Therefore, it is essential for restaurants to maintain high levels of CS to encourage repeat patronage, as satisfied customers are more likely to revisit.

In H5, the relationship between BT and RI was explored. The results (β = 0.684, T = 15.431, *p* < 0.05) confirmed that BT has a significantly positive effect on enhancing customers’ RI. This finding aligns with previous research that suggests that BT significantly influences consumer decision-making by enhancing the perceived safety associated with purchases and the information provided by the brand [93]. It fosters customer loyalty, increases purchase value, and encourages the intention to repurchase, thereby strengthening the relationship between the brand and consumers over time [41].

### 5.2. The Mediating Role of CS and BT and the Moderating Role of OMO

In H6 and H7, this study employed the bootstrap method to examine the mediating effects of CS and BT on the relationship between ESC and RI. The results for H6 (β = 0.076, T = 2.709, *p* < 0.05) indicate that the mediating effect of CS is supported. This finding suggests that ESC can promote RI by enhancing CS [46]. The mediating role of CS in the relationship between service quality and customer loyalty has been confirmed in the hospitality industry, where high-quality service enhances satisfaction, thereby increasing customer loyalty and ultimately facilitating repurchase behavior [94].

In contrast, the results for H7 (β = 0.028, T = 1.450, *p* > 0.05) indicate that the mediating role of BT is not supported. This is attributable to the fact that BT in the restaurant industry fundamentally derives from factors such as food safety transparency, supply chain traceability, and elements directly related to food quality, rather than from single service encounters [91]. This finding is consistent with the earlier results observed in H3, confirming that ESC does not exert a significant positive impact on BT and consequently does not influence RI through BT. For restaurant industry practitioners, these findings imply that efforts to enhance brand recognition and trust should focus on food-related factors, such as ensuring food quality and safety and strengthening supply chain management, rather than solely on improving service encounters.

H8 and H9 examined the moderating effect of the OMO (online–merge–offline) context. The results for H8 (β = 0.002, T = 0.192, *p* > 0.05) and H9 (β = −0.024, T = 1.333, *p* > 0.05) indicate that the hypothesized moderating effects were not supported. According to prior literature, this finding is reasonable, as Generation Z consumers tend to reject standardized, process-driven services and instead seek emotional resonance in their consumption experiences [95]. Additionally, they often perceive AI-driven online data processes as potential infringements on privacy, which can diminish their sense of trust [96].

Consequently, these consumers are less receptive to information “pushed” by enterprises and instead prefer to “pull” information based on their individual interests and preferences [97]. Moreover, the application of OMO scenarios within the restaurant industry is still in its early stages and has yet to effectively align with the specific needs and expectations of Generation Z consumers. Research has further indicated that in situations where employee capabilities are insufficient, the implementation of OMO contexts may exacerbate negative consumer experiences rather than enhance them [98].

### 5.3. Research Implications

This study provides critical insights into the impact of ESC on Generation Z’s CS, BT, and RI within the foodservice industry. The overall findings emphasize several key points, showing that ESC exerts a significant positive influence on CS and RI and demonstrates a mediating effect through CS. However, the influence of ESC on BT, including its indirect pathways, is not significant, indicating that BT is primarily derived from systematic factors rather than single service encounters. Additionally, the OMO context did not exhibit a significant moderating effect, reflecting Generation Z’s heightened sensitivity and caution toward digital service experiences.

From a managerial perspective, this study highlights the pivotal role of ESC in shaping Generation Z consumers’ satisfaction and repurchase behaviors. It underscores the need for targeted training in professional skills and emotional engagement to enhance customer experience and loyalty. The impact of BT further suggests that managers should prioritize systematic improvements such as food quality assurance, supply chain transparency, and risk management mechanisms to build a stable and trustworthy brand image. Although the OMO scenario did not yield a moderating effect in this study, the findings reveal that Generation Z consumers’ expectations for digital services are more nuanced. Companies should focus on enhancing personalization and emotional resonance in OMO service delivery to better align with consumer needs in digitally integrated environments [99].

### 5.4. Limitations and Future Research Directions

Despite the empirical contributions of this study, several limitations should be acknowledged.

First, the sample was drawn from food and beverage outlets located within a limited number of cities. Although the sample covered both restaurant and tea beverage formats, the geographic concentration may constrain the generalizability of the findings. Future research is encouraged to broaden the sampling scope to enhance the representativeness and external validity of the results.

Second, the measurement of service competence primarily relied on customer perception. While this approach enables the direct capture of consumer-side feedback, cross-sectional data based on single consumption episodes are limited in their ability to reflect the dynamic evolution of service competence. Future studies may incorporate multi-source data, such as employee self-assessments and managerial evaluations, to improve the comprehensiveness and robustness of the measurement.

Lastly, the operationalization of the OMO context remains to be further refined. Future research should consider decomposing the OMO construct into specific measurable dimensions—such as the efficiency of information delivery and the depth of online interaction—to more accurately capture its moderating mechanisms and reveal the underlying pathways of influence.

## 6. Conclusions

This study investigates the impact mechanism of ESC on the RI of Generation Z consumers in the foodservice industry. It empirically examines the mediating roles of CS and BT, as well as the moderating role of the OMO context. Based on a quantitative analysis of the sample data, nine hypotheses were proposed, of which five were supported. The results indicate that ESC exerts a direct effect on RI and also functions indirectly via CS. Although BT significantly influences RI, ESC does not exhibit a significant effect on BT or its mediating pathway. Furthermore, the perceived OMO scenario did not demonstrate a statistically significant moderating effect within the current model.

Anchored in the service-dominant logic (SDL) framework, this study incorporates ESC, CS, BT, and RI into a unified analytical model, shedding light on the psychological mechanism by which ESC influences consumption behavior through CS. The findings deepen our theoretical understanding of the “touchpoint–cognition–decision” path in consumer behavior [26]. Additionally, by introducing the OMO context as a moderating variable in a foodservice setting, this research expands the traditional boundaries of service management models under digital transformation and responds to emerging patterns in consumer decision-making in the digital age.

Despite the empirically valuable findings of this study, several limitations remain, including the geographically concentrated sample, single-source data, and insufficient maturity of certain measurement indicators. Future research could expand upon these findings by employing cross-regional sampling, incorporating multi-source data, and conducting studies within more mature OMO environments. Such efforts may contribute to the optimization of service strategies and brand development for food and beverage enterprises targeting Generation Z consumers, while also offering valuable insights for future investigations into service mechanisms, trust formation, and consumer behavior in digital consumption contexts.

## Figures and Tables

**Table 1:** Demographic characteristics of respondents. (*n* = 326).

Item	Option	Frequency	Percentage
Gender	Male	123	37.7
	Female	203	62.3
Age	1995–2000	81	24.8
	2001–2005	224	68.7
	2006–2010	21	6.5
Education Level	High school certificate	10	3.1
	Technical certificate	14	4.3
	Diploma	42	12.9
	Degree	237	72.7
	Others	23	7.0
Daily Consumption Habits	Online	247	75.8
	Offline	79	24.2

**Table 2:** Factor loading.

Constructs	Indicator	Abbreviation Indicator	Loading
Employee Service Capability	The employee is able to accurately answer my questions regarding the product or service.	ESC1	0.784
	The employee is able to recommend appropriate products or services based on my specific needs.	ESC2	0.771
	The employee provides clear and understandable explanations of product usage scenarios.	ESC3	0.803
	The employee listens patiently and understands my specific needs.	ESC4	0.833
	The employee responds promptly to my inquiries during communication.	ESC5	0.768
	The employee makes me feel valued through verbal and non-verbal communication.	ESC6	0.779
	The employee is able to detect my emotional changes and adjust the service accordingly.	ESC7	0.787
	The employee demonstrates empathy during service interactions.	ESC8	0.801
	The employee’s service makes me feel respected and cared for.	ESC9	0.712
	The employee is proficient in operating digital tools.	ESC10	0.803
	The employee is able to synchronize my cross-channel service records using digital tools.	ESC11	0.765
	The employee is able to utilize data analytics to anticipate my potential needs.	ESC12	0.789
Customer Satisfaction	The dishes are fresh and consistent with their descriptions.	CS1	0.935
	The employees demonstrate a friendly and professional service attitude.	CS2	0.882
	The price is aligned with the quality of the food.	CS3	0.875
	The restaurant’s interior design is comfortable and distinctive.	CS4	0.875
	The online ordering and payment process is convenient and efficient.	CS5	0.877
Brand Trust	I believe this restaurant brand delivers product and service quality consistent with its marketing claims.	BT1	0.908
	This restaurant brand adheres to its promises in business operations (e.g., food safety, pricing transparency).	BT2	0.874
	When problems arise, this brand prioritizes customer interests rather than avoiding responsibility.	BT3	0.903
OMO Consumption Scenario	The environment of this restaurant is comfortable and pleasant.	OMO1	0.788
	This restaurant utilizes advanced digital terminals.	OMO2	0.807
	The product information provided online is consistent with that available in the physical store.	OMO3	0.823
	Employees in this restaurant provide services with the assistance of digital devices.	OMO4	0.793
	This restaurant offers me recommendations based on both my online and offline purchase history.	OMO5	0.817
	I can access this restaurant’s services through online channels.	OMO6	0.840
	The restaurant’s online services respond in a timely manner.	OMO7	0.850
Repurchase Intention	I intend to revisit this restaurant in the future.	RI1	0.876
	I would recommend this restaurant to others.	RI2	0.903
	I would say positive things about this restaurant to others.	RI3	0.907
	I will give priority to this restaurant for my next dining occasion.	RI4	0.899

**Table 3:** Cronbach’s alpha, CR, and AVE values.

Constructs	Cronbach’s Alpha	CR	AVE
Employee Service Competence	0.943	0.950	0.614
Consumer Satisfaction	0.934	0.950	0.791
Brand trust	0.876	0.924	0.801
OMO Consumption Scenario	0.917	0.934	0.668
Repurchase Intention	0.918	0.942	0.803

**Table 4:** Discriminant validity of construct Fornell–Larcker criterion.

Constructs	Brand Trust	Repurchase Intention	Consumer Satisfaction	Employee Service Competence	OMO Consumption Scenario
Brand trust	0.895				
Repurchase Intention	0.797	0.896			
Consumer satisfaction	0.299	0.431	0.889		
Employee Service Competence	0.387	0.474	0.312	0.783	
OMO Consumption Scenario	0.814	0.787	0.295	0.398	0.817

**Table 5:** Hypothesis testing.

H	Path	Path Coefficient (β)	Std. Dev. (STDEV)	T Values	*p* Values	Result
H1	ESC -> RI	0.153	0.046	3.319	0.001	Supported
H2	ESC -> CS	0.234	0.082	2.850	0.004	Supported
H3	ESC -> BT	0.041	0.028	1.483	0.138	Not Supported
H4	CS -> RI	0.179	0.052	3.437	0.001	Supported
H5	BT -> RI	0.684	0.044	15.431	0.000	Supported
H6	ESC -> CS -> RI	0.076	0.028	2.709	0.007	Supported
H7	ESC -> BT -> RI	0.028	0.019	1.450	0.147	Not Supported
H8	OMO × ESC -> CS	0.002	0.009	0.192	0.848	Not Supported
H9	OMO × ESC -> BT	−0.024	0.018	1.333	0.183	Not Supported

**Table 6:** Explanatory power.

Predictor	Outcomes	R^2^	f^2^	Q^2^	Fitness of Model
ESC	->CS	0.124	0.051	0.099	GoF = 0.619 SRMR = 0.051 NFI = 0.907
ESC	->BT	0.748	0.005	0.589	
ESC	->CDM	0.692	0.062	0.545	
CS	->CDM		0.091		
BT	->CDM		1.251		

## Data Availability

The original contributions presented in this study are included in the article. Further inquiries can be directed to the corresponding authors.
